# Identification of Diagnostic Genes and Effective Drugs Associated with Osteoporosis Treatment by Single-Cell RNA-Seq Analysis and Network Pharmacology

**DOI:** 10.1155/2022/6830635

**Published:** 2022-09-25

**Authors:** Zhanyue Zhang, Tingbao Zhang, Liangshuang Zhou, Jianzhong Guan

**Affiliations:** ^1^Department of Orthopaedics, The First Affiliated Hospital of Bengbu Medical College, Bengbu 233004, China; ^2^Anhui Provincial Key Laboratory of Tissue Transplantation, Bengbu Medical College, Bengbu 233030, China; ^3^Bengbu Medical College, Bengbu 233000, China

## Abstract

**Background:**

Osteoporosis is a common bone metabolic disease with increased bone fragility and fracture rate. Effective diagnosis and treatment of osteoporosis still need to be explored due to the increasing incidence of disease.

**Methods:**

Single-cell RNA-seq was acquired from GSE147287 dataset. Osteoporosis-related genes were obtained from chEMBL. Cell subpopulations were identified and characterized by scRNA-seq, t-SNE, clusterProfiler, and other computational methods. “limma” R packages were used to identify all differentially expressed genes. A diagnosis model was build using rms R packages. Key drugs were determined by proteins-proteins interaction and molecular docking.

**Results:**

Firstly, 15,577 cells were obtained, and 12 cell subpopulations were identified by clustering, among which 6 cell subpopulations belong to CD45+ BM-MSCs and the other subpopulations were CD45-BM-MSCs. CD45- BM-MSCs_6 and CD45+ BM-MSCs_5 were consider as key subpopulations. Furthermore, we found 7 genes were correlated with above two subpopulations, and F9 gene had highest AUC. Finally, five compounds were identified, among which DB03742 bound well to F9 protein.

**Conclusions:**

This work discovered that 7 genes were correlated with CD45-BM-MSCs_6 and CD45+ BM-MSCs_5 subpopulations in osteoporosis, among which F9 gene had better research value. Moreover, compound DB03742 was a potential inhibitor of F9 protein.

## 1. Introduction

Osteoporosis is a common bone metabolic disease, which is characterized by the loss of bone mass and the disorder of bone structure caused by the imbalance between bone formation and bone resorption. Therefore, patients with osteoporosis are often accompanied by increased bone fragility and fracture rate [1, 2]. Older people and postmenopausal women are particularly susceptible to the disease, which is strongly associated with decreased levels of sex hormones [3, 4]. Among the many clinical adverse consequences of osteoporosis, hip fracture and vertebral fracture are the most serious, and the mortality rate can be as high as 20% after one year of onset. Patients often need hospitalization, and the accompanying complications of other organs are significantly increased, such as pneumonia and pulmonary thrombosis induced by long-term repose [5]. Therefore, effective diagnosis and treatment of osteoporosis are particularly important.

With the proposal of the concept of “precision therapy,” clinical treatment based on sequencing technology has become one of the most important methods to treat cancer [6–8]. The most widely used field of single-cell sequencing technology is tumor research. So far, the analysis of single-cell sequencing data has provided us with a new understanding of the heterogeneity, origin of tumor cells, and occurrence and development of most tumors. In colorectal cancer, Zhang et al. studied the transcriptional map of infiltrating T cell immune receptors; elaborated the subgroup distribution of these cells, tissue distribution characteristics, and tumor heterogeneity; and identified potential drug targets [9]. Bian et al. used this technology to study single cell copy number variation during the occurrence and metastasis of human colorectal cancer and analyzed abnormal DNA methylation and differential expression [10]. In breast cancer, researchers discovered and identified the polyclonal origin of breast cancer by means of cell trajectory inference and tumor heterogeneity analysis, providing a new theoretical basis for the early diagnosis of breast cancer [11]. By single-cell sequencing of microenvironment cells, the researchers mapped a variety of immune cells infiltrated in the breast cancer microenvironment [12–14]. However, the heterogeneity, diagnosis, and treatment of osteoporosis are rarely studied by single cell sequencing.

In view of the powerful function of single-cell sequencing technology in tumor research, this study will use this technology to explore osteoporosis and provide new ideas for early diagnosis and treatment of osteoporosis through single-cell sequencing analysis.

## 2. Materials and Methods

### 2.1. Data Acquisition

Single-cell sequencing data GSE147287 [15], downloaded from the Gene Expression Omnibus (GEO) dataset, includes two samples (osteoporosis patients and osteoarthritis patients). First of all, from the PubChem database (https://pubchem.ncbi.nlm.nih.gov/) [16], to download 3D/2D structure of osteoporosis drugs, mainly including of glucocorticoid hormones and calcineurin inhibitors, chEMBL (http://www.ebi.ac.uk/chembl/) [17] was used to download these drugs active site-related genes.

### 2.2. Data Control

The R Package Seurat function [18] was used to set the expression of each gene in at least 3 cells, and each cell expressed at least 250 genes to filter a single cell. Mitochondria and rRNA quantities were further calculated by PercentageFeatureSet function. Genes expressed in each cell were less than 5,000, the percentage of mitochondria was less than 25%, and the UMI of each cell was at least greater than 100. The FindVariableFeatures function was employed to detect highly variable genes, followed by scaling and PCA dimensionality reduction for all genes using the ScaleData function.

### 2.3. Cell Type Annotation

We used FindNeighbors and FindClusters [19] (Dim = 20, Resolution = 0.1) here for cell clustering. The FindAllMarkers function was conducted to select the marker gene. Kyoto Encyclopedia of Genes and Genomes (KEGG) pathway annotation was performed using R Package ClusterProfiler [20].

### 2.4. Compared Patient with and without Osteoporosis

Differential gene expression analysis was performed in patients with and without osteoporosis in the GSE35959 dataset [21] by limma package [22] with |log2(Fold Change)| > 1 and *p* < 0.05.

### 2.5. Classification Algorithms

In GSE35959 dataset, RMS was used to construct diagnostic model [23]. We combined multiple genes model as well as single gene model to identify osteoporosis.

### 2.6. Computation of Estimate Score, Stromal Score, and Immune Score

R software estimation of stromal and immune cells in malignant tumors using expression data (ESTIMATE) arithmetic [24] was utilized to compute overall stroma level (stromal score), the immunocyte infiltration (immune score), and the combination (ESTIMATE score).

### 2.7. ssGSEA

For analyzing the infiltration level of 28 immune cells, we used the single-sample gene set enrichment analysis (ssGSEA) method of R software gene set variation analysis (GSVA) [25] package [26].

### 2.8. Molecular Docking Simulation

In this experiment, Autodock Vina software [27] was used for molecular docking simulation. AutoDockTools 1.5.6 [28] is used to prepare all input files. PDB-IDs of target genes were obtained from Protein Data Bank (PDB) [29] database. To identify the most binding mode for ligand molecules, the Lamarckian algorithm [30] was used with exhaustiveness being set to 8, the maximum number of conformations output being set to 10, and the maximum allowable energy range being set to 3 kcal/mol. The results were processed by Pymol [31]. 100 ns molecular dynamics simulations were performed using Gromacs2019 software package [32] to assess the binding stability of the receptor-ligand complex.

## 3. Results

### 3.1. Single-Cell RNA-Seq Profiling and Clustering

First, the single-cell data was filtered by setting each gene to be expressed in at least three cells, with at least 250 genes per cell, yielding 17,669 cells. The quality control diagram of samples before and after filtration is shown in Figure S1A-B, which requires that the detected cells express less than 5,000 genes, the mitochondrial content is less than 25%, and the UMI of each cell is at least more than 100. Therefore, 15,577 cells are finally obtained. In addition, a significant positive relationship was observed between UMI and the number of detected genes (Pearson's *r* = 0.73), and there was a significant negative correlation between UMI and mitochondrial content (Pearson's *R* = −0.16), gene number, and mitochondrial content (Pearson's *R* = −0.16) (Figure S1C). The cells were mapped to two dimensions based on PC_2 and PC_1 groups. It has been found that the two cell subpopulations were not significantly separated (Figure S1D).

**Figure 1:**
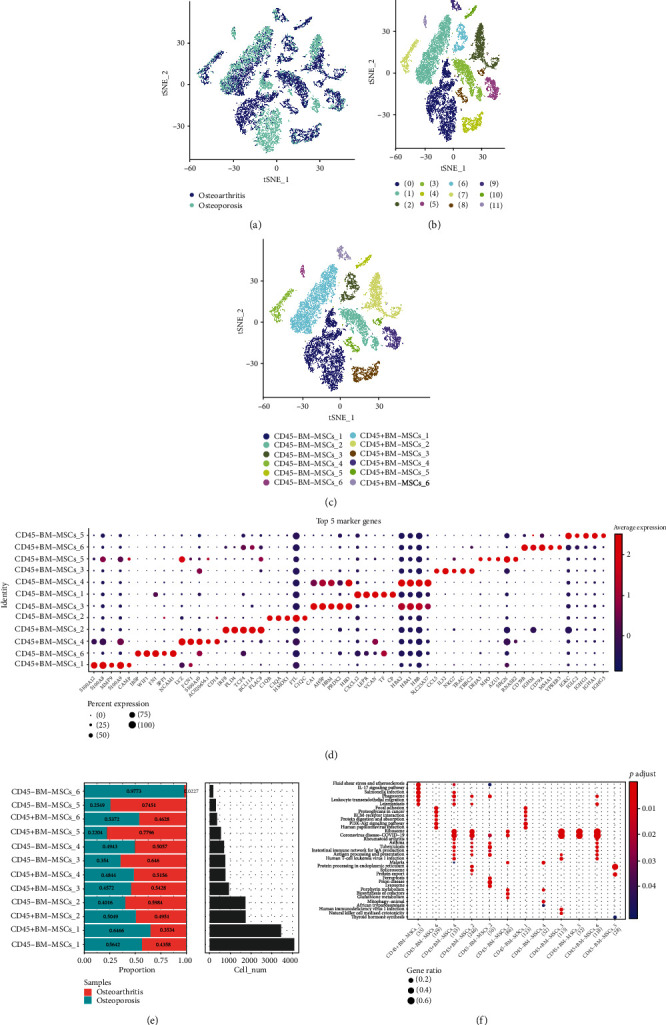
Single-cell RNA analysis of osteoporosis patients and osteoarthritis patients. (a) t-Distributed stochastic neighbor embedding of two samples. (b) t-Distributed stochastic neighbor embedding of 12 subpopulations. (c) t-Distributed stochastic neighbor embedding of 12 subpopulations after BM-MSCs cell annotation. (d) The proportion and cell numbers of 12 subpopulations in two samples. (f) Kyoto Encyclopedia of Genes and Genomes enrichment analysis of 12 subpopulations.

Furthermore, t-distributed stochastic neighbor embedding (t-SNE) algorithm, which is commonly in visualizing high dimensional data, was applied here for cell population clustering.  Figure 1(a) is the t-SNE diagram of two samples, and Figure 1(b) is t-SNE of 12 cell subpopulations. Next, we detected the expression of CD45 in 12 subpopulations and found that 6 subpopulations were CD45+ BM-MSCs and 6 subpopulations were CD45-BM-MSCs (Figure 1(c), Figure S1E).

Accordingly, we performed differential analysis using the FindAllMarkers function with logfc = 0.5, Minpct = 0.5, and adjPval < 0.05. The top 5 differential genes of 12 cell subpopulations in the heat map plot are illustrated in Figure 1(d). The distribution of 12 cell subpopulations in two samples is shown in Figure 1(e). KEGG analysis indicated that 35 pathways were significantly enriched on 12 cell subpopulations, and many of them were related to tumorigenesis (Figure 1(f)).

### 3.2. Identification of Hub Genes

Fisher test was adopted to analyze the distribution variation of 12 cell subpopulations between osteoporosis and osteoarthritis, as the cutoff of FC>4 or FC<0.25 and*p* < 0.05, and subpopulations of CD45- BM-MSCs_6 and CD45+ BM-MSCs_5 were selected as vital for next analysis (Table 1).

**Figure 2:**
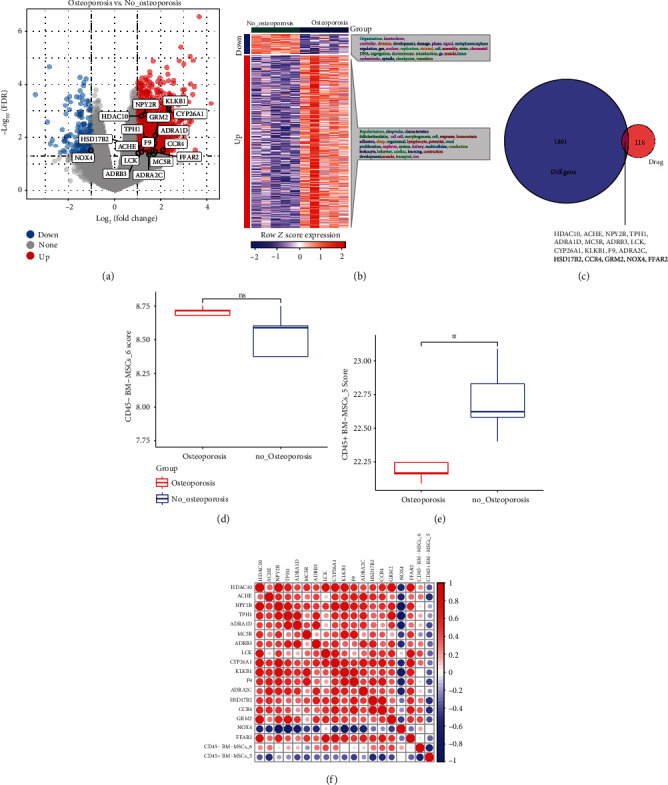
Identification of hub genes. (a) Volcano plot of differentially expressed genes between osteoporosis and nonosteoporosis. (b) Heatmap of differentially expressed genes between osteoporosis and nonosteoporosis. (c) Venn of differentially expressed genes and target genes for osteoporosis drugs. (d) CD45-BM-MSCs score differences analysis between osteoporosis and nonosteoporosis. (e) CD45+BM-MSCs score differences analysis between osteoporosis and nonosteoporosis. (f) The correlation analysis between 17 hub genes and CD45-BM-MSCs subpopulation or CD45+BM-MSCs subpopulation.

Next, limma package was used to identified differentially expressed gene between osteoporosis and no-osteoporosis; as a result, 191 downregulated genes and 1,717 upregulated genes were screened (Figures 2(a) and 2(b)). Venn analysis between 1,908 differentially expressed gene and 135 genes, which from chEMBL, showed that only 17 genes were targets for the treatment of osteoporosis (Figure 2(c)).

Based on the above marker genes of subpopulations, ssGSEA analysis was used to calculate score of the two hub subpopulations, and variance analysis of subpopulations score between osteoporosis and no-osteoporosis indicated that CD45+ BM-MSCs_5 subpopulation had significant difference (Figures 2(d) and 2(e)). The correlation analysis of the 17 genes with the two hub subpopulations score demonstrated that 7 genes (HSD17B2, ACHE, CCR4, F9, ADRA1D, MC5R, and GRM2) were negatively correlated with CD45+ BM-MSCs_5 subpopulation (Figure 2(f)).

### 3.3. Construction of Diagnostic Model Base on 7 Genes

**Figure 3:**
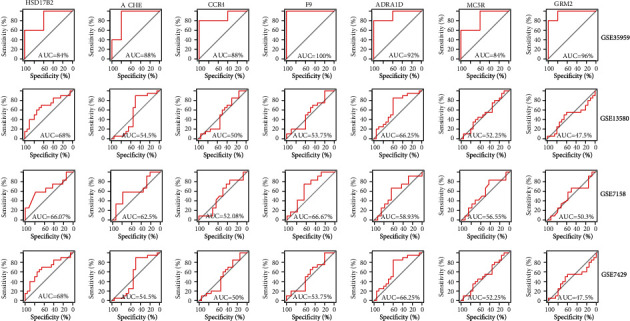
Individual ROC curves for 7 genes in GSE35959, GSE13580, GSE7158, and GSE7429 dataset.

**Figure 4:**
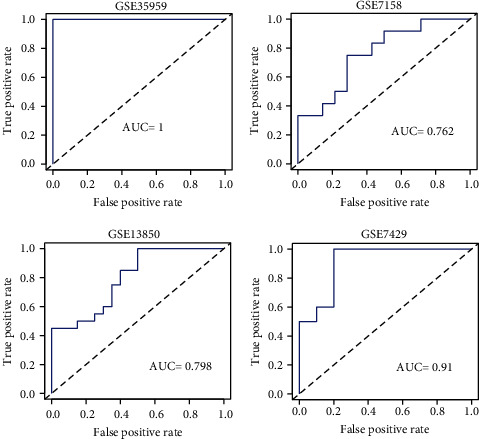
Construction of 7 key gene diagnostic models.

The diagnostic models were separately constructed for these 7 genes. The results showed that the AUC values calculated by these 7 genes alone were not well (Figure 3). Due to the poor diagnosis of single genes, rms was used to construct diagnostic model based on 7 genes, and the AUC values in GSE35959 dataset, GSE7158 dataset, GSE13850 dataset, and GSE7429 dataset, were, respectively, 1, 0.762, 0.798, and 0.91 (Figure 4), which are better than the AUC values of singles genes. This data indicated that the combination of these 7 genes is of great significance in diagnosing osteoporosis patients.

### 3.4. Analysis of Hub Genes and Immune

**Figure 5:**
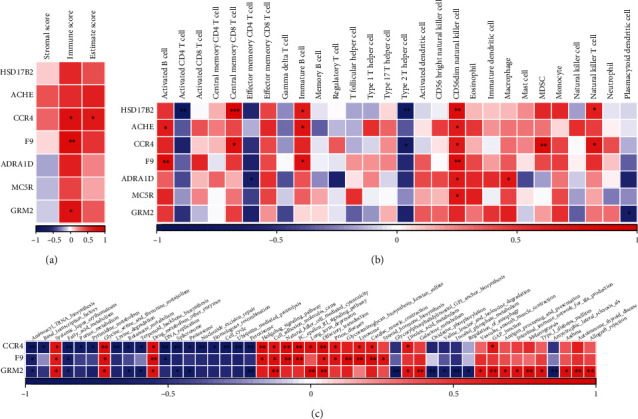
The correlation analysis between hub genes and immune. (a) The correlation analysis between 7 hub genes and immune scores. (b) The correlation analysis between 7 hub genes and 28 immune cells scores. (c) Heatmaps of potentially regulated pathways of 3 genes associated with immune.

First, stromal score, immune score, and ESTIMATE score were calculated by ESTIMATE, and the results showed that three genes (CCR4, F9, and GRM2) were positively correlated with the immune score (Figure 5(a)). Then, 28 characteristic genes of immune cells obtained from previous study were used to calculate the score of immune cells by ssGSEA method. Pearson correlation analysis showed that CCR4, F9, and GRM2 were significantly associated with some immune cells (Figure 5(b)). Enrichment analysis was conducted by GSVA package, and the correlation between three genes and pathways was calculated by Pearson's method. The results indicated that these three genes were correlated with multiple pathways (Figure 5(c)).

### 3.5. Prediction of Therapeutic Drugs

**Figure 6:**
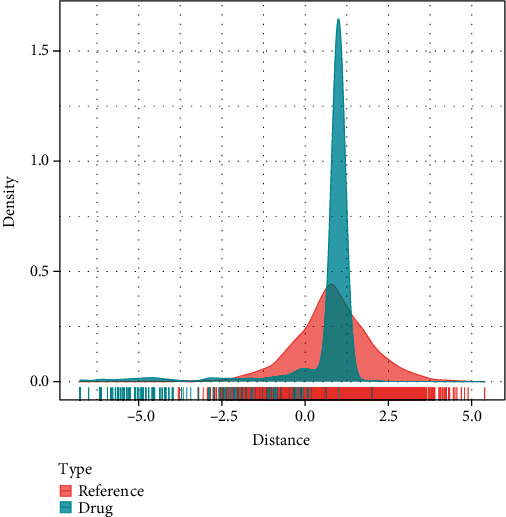
Distance density scaling diagram of drug to F9-related gene sets.

Since the area under the curve (AUC) values of F9 gene were higher than those of the other two genes in multiple data sets, we believed that the role of F9 gene in osteoporosis patients has more potential to be studied. The RCORR function of Hmisc package was used for correlation analysis of osteoporosis patients in GSE35959 data set, and a total of 1377 genes were highly significantly correlated with F9 gene. The distance density plot of drug to F9-related gene set is shown in Figure 6.

**Figure 7:**
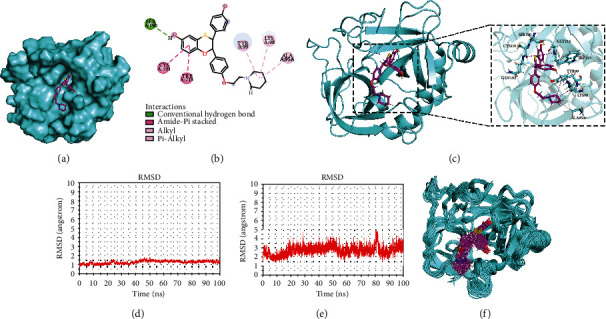
Molecular docking and dynamics simulation. (a) Surface binding of compound DB03742 to F9 protein. (b) 2D diagram of interaction between compound DB03742 and F9 protein. (c) 3D diagram of interaction between compound DB03742 and F9 protein. (d) RMSD of F9 protein skeleton during 100 ns molecular dynamics simulation. (e) RMSF of F9 protein skeleton during 100 ns molecular dynamics simulation. (f) Binding diagram of compound DB03742 to F9 protein during 100 ns molecular dynamics simulation.

Molecular docking was conducted to verify whether the top 5 compounds closest to F9 gene set (Table 2) had significant regulatory effects on F9 protein. The results showed that all the key compounds in the network, especially DB03742, had strong affinity for F9 protein (-8.2 kcal/mol) (Figure 7(a)). In addition, DB03742 could form a stable complex with F9 by hydrogen bonding with SER190 of F9 protein and hydrophobic interaction with ALA95, LYS98, TYR99, CYS191, and TRP215 (Figures 7(b) and 7(c)).

Molecular dynamics simulations at 100 ns showed that the F9 protein concept was stable (Figures 7(d) and 7(f)). In addition, the RMSF value of compound DB03742 was basically stable at about 3 Å (Figure 7(e)). In general, compound DB03742 binds to the active site of F9 protein relatively stable, suggesting that compound DB03742 has a high potential as an inhibitor of F9 protein.

## 4. Discussion

ScRNA-seq is a highly useful tool in transcriptional classification of various disease cell types [33–36]. Herein, osteoporosis scRNA-seq data from GEO database was collected to distinguish cell subpopulations, and here, we determined 12 subgroups, among which 6 cell subpopulations belong to CD45-BM-MSCs. In a large set of samples, specifically expressed gene markers could be utilized as specific markers in cell subgroup identification. Furthermore, 7 key genes associated with osteoporosis treatment drugs were screened and found to be negatively correlated with CD45+ BM-MSCs_5 subgroup score, and they showed excellent diagnosis efficiency, especially F9 gene. Through network pharmacology, we found that DB03742 is highly bound to F9 protein, which is a potential F9 protein inhibitor, and speculated that DB03742 may be effective in the treatment of osteoporosis. In previous research, Liang et al. reported the heterogeneity of tumor immune cells analyzed by scRNA-seq, and a risk model was constructed to predict the survival of ovarian cancer samples [37]. Juan Lu *et al*. analyzed the heterogeneity of the TIME at the single-cell level and determined a 3-gene model that could accurately evaluate the survival outcome and immunotherapy response of HCC samples [38]. This work was the first to identify the heterogeneity of osteoporosis at the single-cell level and to identify key genes and therapeutics for the diagnosis of osteoporosis.

Here, we performed differential analysis on gene expression data from the GSE35959 database. 7 genes were vital for diagnosis of osteoporosis, among which 17*β*-hydroxysteroid dehydrogenase type 2 (17*β*-HSD2) is an enzyme that catalyzes the conversion of estradiol (E2) and testosterone (T) to estrone (E1) and androstenedione, respectively. Blockade of 17*β*-HSD2 increases intracellular E2 and T, inhibits further bone resorption by osteoclasts, and stimulates osteoblast osteogenesis by estrogen and androgen receptor stimulation, respectively [39, 40]. A minor reduction in Th2 cells was detected from the mice with CCR4 knock-out in parallel with major Treg migration impairment. Study showed that such a phenomenon was related to higher proinflammatory and osteoclastogenic cytokine levels and increased inflammatory bone loss [41]. At present, most genes have not been reported to play a role in osteoporosis. Moreover, the prediction performance of 7 genes model was not very good, probably because of the sample size. These results suggested that these genes may play an important role in the development of osteoporosis and provided evidences for further research.

In this work, we analyzed the gene expression profile of the scRNA-seq data, the results of which improved our understanding of the heterogeneity of osteoporosis at the single-cell level and provided a 7-gene-based diagnostic model and therapeutic agent. However, there are some limitations to this study. First, the sample size was relatively small. Secondly, functional experiments and potential molecular mechanisms of these 7 genes need to be studied.

By the analysis of scRNA-seq, we analyzed the heterogeneity of osteoporosis at the single-cell level and determined a 7-gene diagnostic model that could accurately diagnostic patients with osteoporosis.

## Figures and Tables

**Table 1:** Differential analysis of cell types.

Osteoporosis/osteoarthritis		
cell_name	*p*.val	fc
CD45+ BM-MSCs_1	1.03E-64	1.952078384
CD45- BM-MSCs_6	1.29E-43	40.55324249
CD45+ BM-MSCs_4	0.045726035	0.861342268
CD45+ BM-MSCs_2	0.185992587	0.934653196
CD45- BM-MSCs_2	1.50E-25	0.584120062
CD45- BM-MSCs_3	5.45E-21	0.489261845
CD45- BM-MSCs_1	3.69E-11	1.274037866
CD45- BM-MSCs_4	0.175479663	0.898346627
CD45+ BM-MSCs_3	8.77E-05	0.765464953
CD45+ BM-MSCs_5	1.89E-48	0.249892213
CD45+ BM-MSCs_6	0.52377383	1.073767195
CD45- BM-MSCs_5	1.76E-21	0.309302402

**Table 2:** Molecular docking of drugs to F9 protein.

Compound	Autodock Vina score	H-bond interactions	Hydrophobic interactions
DB00269	-6.4	LYS98, SER195	TYR99
DB00947	-7.7	LYS98, SER190	LYS98, TYR99, SER190, CYS191, SER214, TRP215, CYS220
DB01357	-7.3	SER190	SER190, CYS191, TRP215, CYS220
DB02715	-8.0	SER190	TYR99, CYS191, TRP215,
DB03742	-8.2	SER190	ALA95, LYS98, TYR99, CYS191, TRP215

## Data Availability

The datasets analyzed in this study could be found in GSE35959 at [https://www.ncbi.nlm.nih.gov/geo/query/acc.cgi?acc=GSE35959], GSE7158 dataset at [https://www.ncbi.nlm.nih.gov/geo/query/acc.cgi?acc=GSE7158], GSE13850 dataset at [https://www.ncbi.nlm.nih.gov/geo/query/acc.cgi?acc=GSE13850], and GSE7429 dataset at [https://www.ncbi.nlm.nih.gov/geo/query/acc.cgi?acc=GSE7429].

## Supplementary Materials

Supplementary Materials Figure S1. Clustering and dimension reduction analysis of single cell data. A: Correlation analysis of UMI and number of mRNA, Mitochondrial gene. B: MRNA/UMI/mitochondrial content/rRNA content of samples before filtration. C: MRNA/UMI/mitochondrial content/rRNA content of samples after filtration. D: The sample distribution of PCA dimension reduction and the anchor point diagram of PCA. E: CD45 expression of 12 subpopulations.
