# Supplementary figures and images for: Identification of Diagnostic Genes and Effective Drugs Associated with Osteoporosis Treatment by Single-Cell RNA-Seq Analysis and Network Pharmacology
Source: Mediators Inflamm. 2022 Sep 25;2022:6830635. doi: 10.1155/2022/6830635 (PMC9527401; doi:10.1155/2022/6830635)

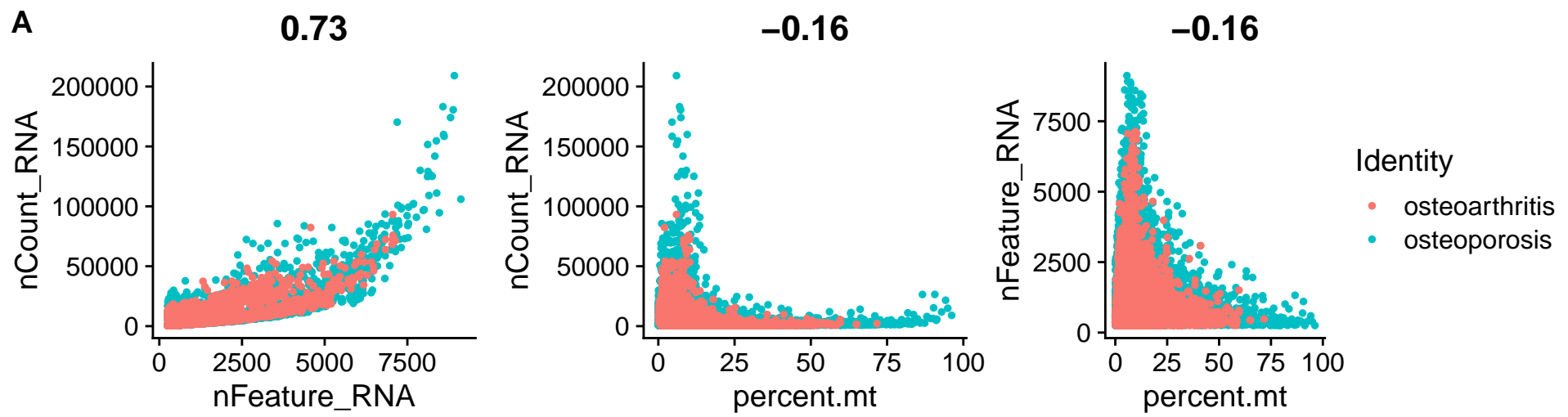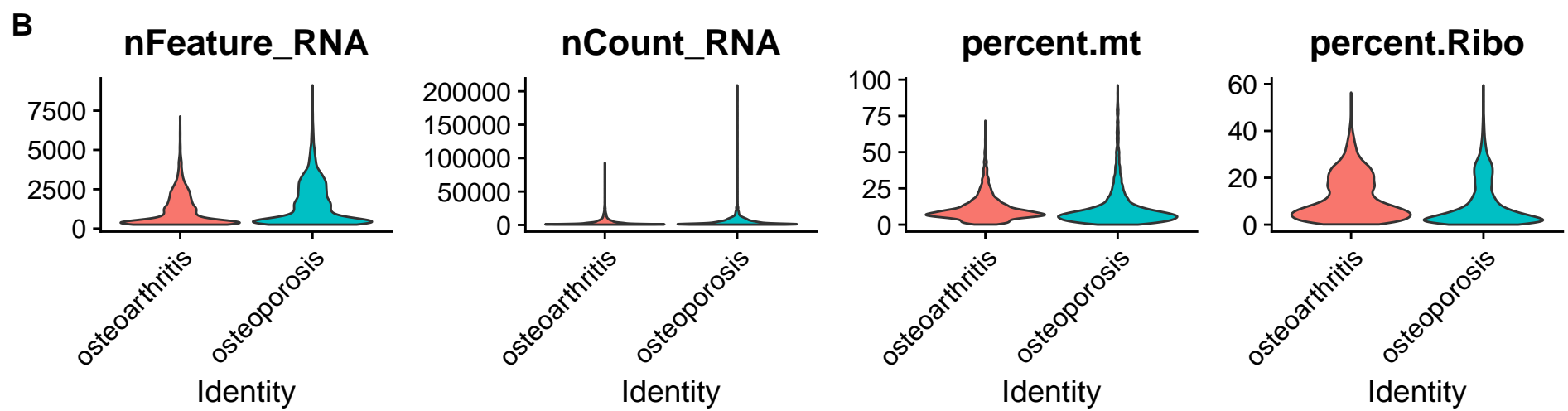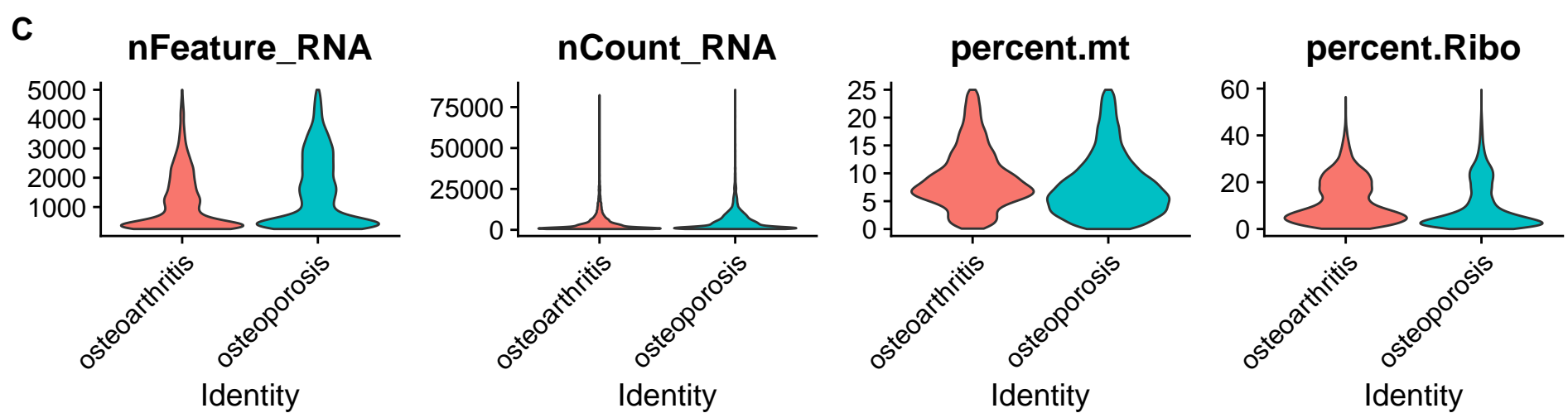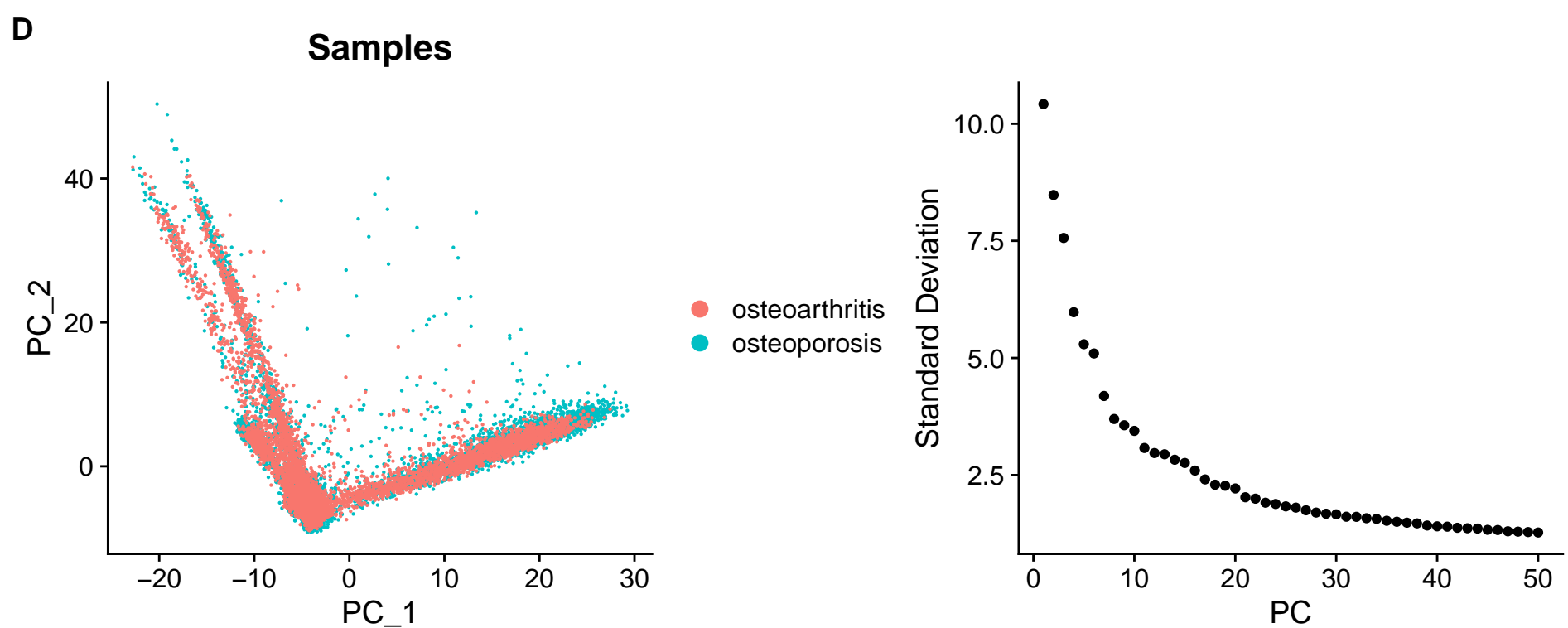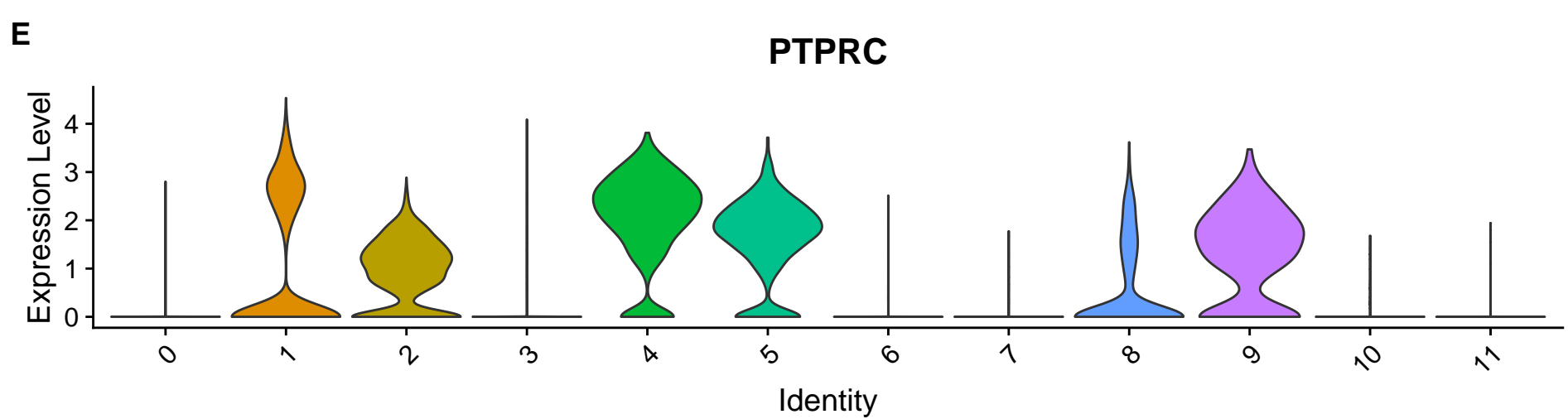

Supplement: Supplementary Materials — Figure S1. Clustering and dimension reduction analysis of single cell data. A: Correlation analysis of UMI and number of mRNA, Mitochondrial gene. B: MRNA/UMI/mitochondrial content/rRNA content of samples before filtration. C: MRNA/UMI/mitochondrial content/rRNA content of samples after filtration. D: The sample distribution of PCA dimension reduction and the anchor point diagram of PCA. E: CD45 expression of 12 subpopulations. [file 6830635.f1.pdf]
